# A Data Encryption Solution for Mobile Health Apps in Cooperation Environments

**DOI:** 10.2196/jmir.2498

**Published:** 2013-04-25

**Authors:** Bruno M Silva, Joel JPC Rodrigues, Fábio Canelo, Ivo C Lopes, Liang Zhou

**Affiliations:** ^1^Instituto de Telecomunicações, University of Beira InteriorCovilhãPortugal; ^2^Nanjing University of Posts and TelecommunicationsNanjingChina

**Keywords:** mobile health, mHealth, mobile computing, eHealth, cooperation, encryption, security

## Abstract

**Background:**

Mobile Health (mHealth) proposes health care delivering anytime and anywhere. It aims to answer several emerging problems in health services, including the increasing number of chronic diseases, high costs on national health services, and the need to provide direct access to health services, regardless of time and place. mHealth systems include the use of mobile devices and apps that interact with patients and caretakers. However, mobile devices present several constraints, such as processor, energy, and storage resource limitations. The constant mobility and often-required Internet connectivity also exposes and compromises the privacy and confidentiality of health information.

**Objective:**

This paper presents a proposal, construction, performance evaluation, and validation of a data encryption solution for mobile health apps (DE4MHA), considering a novel and early-proposed cooperation strategy. The goal was to present a robust solution based on encryption algorithms that guarantee the best confidentiality, integrity, and authenticity of users health information. In this paper, we presented, explained, evaluated the performance, and discussed the cooperation mechanisms and the proposed encryption solution for mHealth apps.

**Methods:**

First, we designed and deployed the DE4MHA. Then two studies were performed: (1) study and comparison of symmetric and asymmetric encryption/decryption algorithms in an mHealth app under a cooperation environment, and (2) performance evaluation of the DE4MHA. Its performance was evaluated through a prototype using an mHealth app for obesity prevention and cares, called SapoFit. We then conducted an evaluation study of the mHealth app with cooperation mechanisms and the DE4MHA using real users and a real cooperation scenario. In 5 days, 5 different groups of 7 students selected randomly agreed to use and experiment the SapoFit app using the 7 devices available for trials.

**Results:**

There were 35 users of SapoFit that participated in this study. The performance evaluation of the app was done using 7 real mobile devices in 5 different days. The results showed that confidentiality and protection of the users’ health information was guaranteed and SapoFit users were able to use the mHealth app with satisfactory quality. Results also showed that the app with the DE4MHA presented nearly the same results as the app without the DE4MHA. The performance evaluation results considered the probability that a request was successfully answered as a function of the number of uncooperative nodes in the network. The service delivery probability decreased with the increase of uncooperative mobile nodes. Using DE4MHA, it was observed that performance presented a slightly worse result. The service average was also slightly worse but practically insignificantly different than with DE4MHA, being considered negligible.

**Conclusions:**

This paper proposed a data encryption solution for mobile health apps, called DE4MHA. The data encryption algorithm DE4MHA with cooperation mechanisms in mobile health allow users to safely obtain health information with the data being carried securely. These security mechanisms did not deteriorate the overall network performance and the app, maintaining similar performance levels as without the encryption. More importantly, it offers a robust and reliable increase of privacy, confidentiality, integrity, and authenticity of their health information. Although it was experimented on a specific mHealth app, SapoFit, both DE4MHA and the cooperation strategy can be deployed in other mHealth apps.

## Introduction

In the last decade, health telematics, also known as electronic health (eHealth), have offered patients major improvements in their lives by providing more accessible and affordable health care solutions [1,2]. This is particularly true for patients that live in remote rural areas, travel constantly, are physically incapacitated, elderly, or chronically ill. Telemedicine assumes the use of medical information, also known as electronic health records (EHRs), exchanged via electronic communications improving the patients’ health status [3]. The rapid evolution of information and communication technology (ICT) infrastructures enables and provides rapid access to patient data. The Web 2.0 concept and the emerging Web 3.0 offer opportunities to health care professionals never seen before [4,5]. Now, physicians can perform many tasks through these modern technologies, such as (1) sharing medical videos, photos, and presentations (via YouTube, Flickr, and Slideshare, respectively), (2) use blogs to post medical cases and images, (3) share hospital management information, (4) use social networking to share medical ideas and tasks, and (5) use RSS feeds to keep track of alerts on specific medical interests.

With the advent of mobile communications using smart mobile devices that support 3G and 4G mobile networks for data transport, mobile computing has been the main attraction of research and business communities, thus offering innumerous opportunities to create efficient mobile health solutions. Mobile health (mHealth) is the new edge on health care innovations. It delivers health care anywhere and anytime, surpassing geographical, temporal, and even organizational barriers [6,7]. Laxminarayan and Istepanian defined mobile health for the first time in 2000, as “unwired e-med*”* [8]*.* In 2003, the term “mHealth” was defined as the *”*emerging mobile communications and network technologies for health care systems*”* [9]. Laxminarayan et al, in 2006, presented a comprehensive study on the impact of mobility on the existing eHealth commercial telemedical systems. They also presented other relevant computing and information technologies that will influence and offer the basis for the next generation of mHealth services [10]. Furthermore, this study served as the basis for future studies on mHealth technologies and services [11]*.* Several research topics related to health have gathered important findings and contributions using mHealth, such as cardiology [12,13], diabetes [14-16], obesity [17-20], and smoking cessation [21]. More specifically, mHealth apps were applied to health monitoring, disease prevention and detection, basic diagnosis, and in more advanced services. mHealth services are also becoming popular in developing countries where health care facilities are frequently remote and inaccessible [2,22].

Mobile devices and wireless communications present several challenging characteristics and constraints, such as battery and storage capacity, broadcasting constraints, signal interferences, disconnections, noises, limited bandwidths, and network delays. In this sense, cooperation-based approaches are presented as a solution to solve such limitations, focusing on increasing network connectivity, communication rates, and reliability.

In this paper, we present a data encryption solution for mHealth apps (DE4MHA) in cooperative environments guaranteeing data confidentiality, integrity, and authenticity. This novel and early-proposed cooperation strategy [23] for mHealth apps focuses on forwarding and retrieving data to and from nodes that have no direct connection to an mHealth service. In this way, devices without Internet connectivity can use mHealth apps without problems. This cooperation approach presents a reputation-based strategy where a Web service manages the access control and the cooperation among nodes along with their reputation. It considers the following three main components: a *node control message,* a *requester control message,* and a *cooperative Web service* (CWS). Both control messages are used to manage a local cooperation between two or more nodes. The CWS includes a reputation table for all the nodes and decides which nodes can have access to the requested services. The cooperation strategy and the DE4MHA was deployed and evaluated in an mHealth app for obesity prevention and control, called SapoFit [24-26]. To the best of our knowledge, there are no cooperative solutions thus far for mHealth services and apps considering this network scenario with constant network disconnection. DE4MHA uses symmetric and asymmetric encryption and decryption techniques. We used the Rivest, Shamir, Adleman (RSA) algorithm [27] for asymmetric encryption/decryption to ensure key exchange confidentiality, and the Advanced Encryption Standard (AES) [28] algorithm for symmetric encryption/decryption for data confidentiality. To ensure data integrity, we have created a message digest that creates a hash of transmitted data. For data authenticity, we used a digital signature. We encrypted the hash message with the RSA private key. To secure the communication with the SapoFit Web service (WS), we used the Hypertext Transfer Protocol Secure (HTTPS) protocol.

In this paper we report two studies that were performed to design and construct the DE4MHA algorithms: (1) a direct evaluation and comparison of several encryption algorithms, and (2) a series of trials evolving 35 people and 7 different mobile devices with SapoFit. The first study revealed what algorithms performed best in an mHealth app in cooperation environments. Overall, this study evaluated the performance of the DE4MHA over the cooperation mechanisms for mHealth apps. The second study revealed that real users experimenting on the SapoFit app trusted DE4MHA. More relevant, this study concluded that the performance of the app used was not affected by the inclusion of DE4MHA.

## Methods

### Overview

This study used an existing mHealth app, called SapoFit, to deploy, evaluate, and validate the proposed solution. This app uses a cooperation strategy that addresses two related limitations to mHealth apps with service-oriented architectures, namely the network infrastructure and Internet connectivity dependency. It follows a reputation-based approach as an incentive method for cooperation, which includes a Web service to manage all the network cooperation. It is responsible for verifying the cooperation status of neighbor nodes and to provide relay nodes the required data in order to perform a full data request.

### Cooperation Strategy for mHealth Apps

The cooperation strategy for mHealth apps with service oriented architectures (SOAs) is based on the following two mobile modules and one remote module, respectively: (1) the *node control message,* (2) the *requester control message*, and (3) the *CWS*.

The mobile *nodes control messages* aim to provide an awareness of the relay node status, that is, if the node is willing to cooperate and in what conditions. It contains the established node unique identifier, the battery state, the Internet connectivity status, and the cooperation status (ie, if it is cooperative or not).

The *requester control message* is sent by the initial requester node first (the mobile device with mHealth app requesting health data), and it comprises the following five main components: (1) the requester ID, the node unique identifier, (2) the service request, that is, what the node is specifically requesting (eg, the login token or its health profile), (3) the neighbors list, (4) the reputation list, and (5) the achieved cooperation time (ACT).

The *CWS* is responsible for performing a fair access control to data. Thus, according to the received reputation information, the Web service holds the final reputation list in order to decide if a requester node should have access to the mHealth app Web service or not. The reputation list contains all registered network nodes with their identifier and their corresponding reputation value.

**Figure 1:**
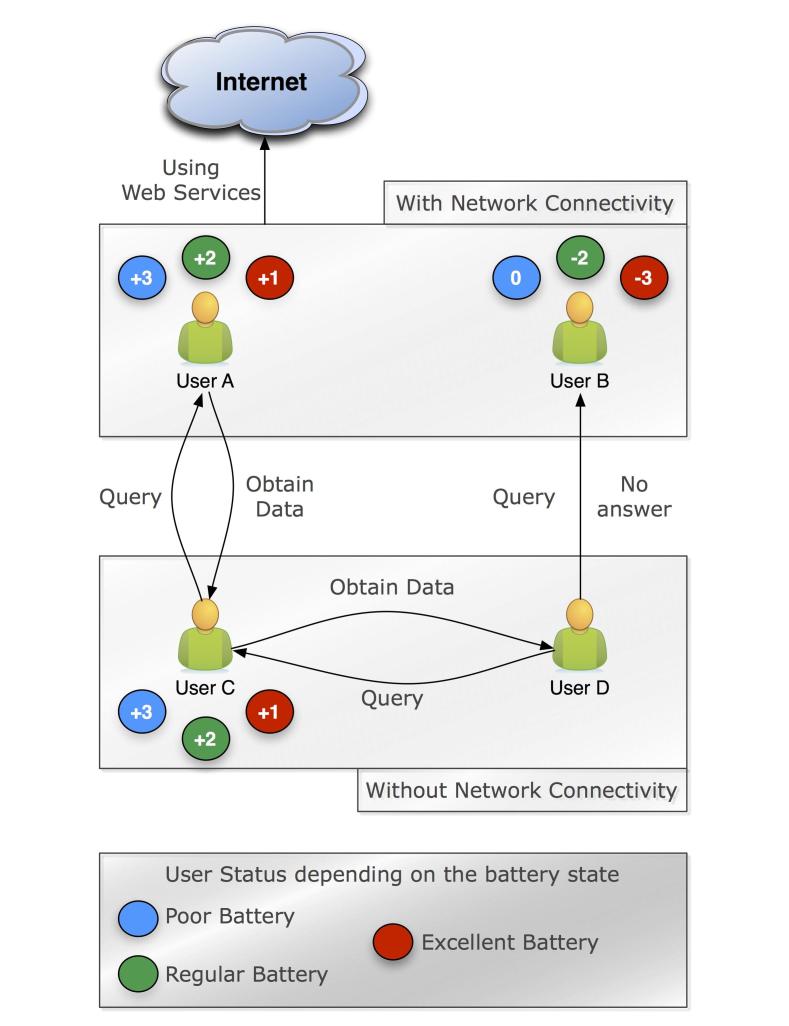
Illustration of the interaction for an mHealth app with the proposed cooperation approach for 4 users.

Figure 1 presents a user scenario of the mHealth cooperation approach. *User A* has network connectivity and cooperates. *User B* has network connectivity and does not cooperate. The status value is according to the battery status. Then, the status value will suffer a negative impact according to the battery status. *Users C* and *D* do not have network connectivity. *User C* queries *User A* for cooperation and receives a positive response and all the requested data. *User D* queries *User B* for cooperation and receives a negative response. Then, *User D* requests data from *User C* that answers this request, getting positive status by cooperating.

### SapoFit App

**Figure 2:**
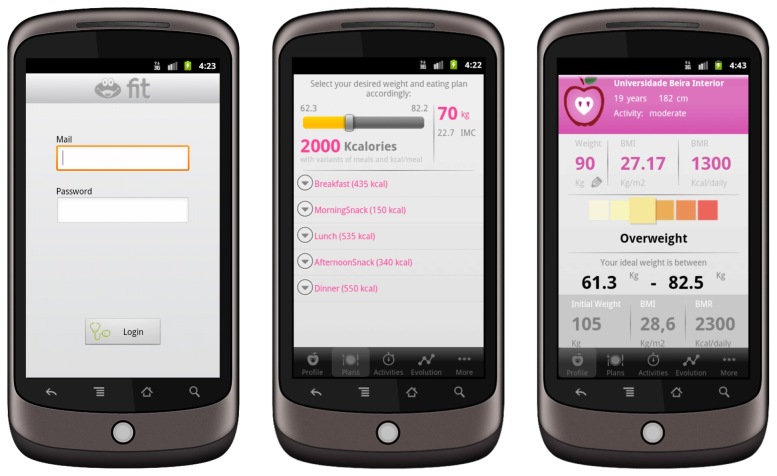
Screenshots of the three main activities of SapoFit app: Login, Plans, and User Profile.

SapoFit is a weight control mobile app that allows users to keep track of weight in a healthier and more practical way. SapoFit allows users to control their weight, body mass index (BMI), basal metabolic rate (BMR), sports activity, and the possibility to follow food plans based on their needed calories. In this mHealth app, all the users must be registered in a Web service. Figure 2 presents screenshots of three main activities of the SapoFit app: *Login, Plans,* and *User Profile.*

Cooperating nodes have a better reputation, and have priority over selfish nodes to access the mHealth app services.

### Data Encryption Algorithm for Mobile Health Apps (DE4MHA)

The process begins with a mobile node (a person using SapoFit) trying to access the SapoFit Web Service that contains the user profile, weight measures, fitness, and diet indications.

A SapoFit user (mobile requester node) without network connectivity and therefore without access to the SapoFit WS obtains the required health information through cooperation. Another SapoFit user with network connectivity (mobile requested node) will forward the requested health information from the SapoFit Web service. Both the requested and requester nodes will create a pair of RSA keys and send public keys to both the requested and requester node through Bluetooth. After the public key exchange, the requested node creates an AES session key.

The next step is the creation of the digest message and its encryption using the private key. The Message Digest 5 (MD5) algorithm was used to create a 128-bit hash. For data authenticity, we used a digital signature. A digital signature is created for the message containing requested health information. This digital signature allows any node to verify that the message is the original one. By decrypting the digital signature with the public key, the original digest message is obtained. The receiver node then creates a new hash of the received message and compares it to the decrypted digest message to guarantee authenticity. The digital signature is then added to the message. When the message containing the session key is received, if its integrity and authenticity is verified, the requester node then sends an acknowledgement (*ack*) to the requested node. This method guaranties safe communication between nodes; if the integrity and authenticity is not verified, the communication between nodes is ended.

A mobile node with network connectivity will access the cooperative WS to obtain the required health information. To secure all communication with the WS the Secure Socket Layer (SSL) over the HTTP (also known as HTTPS) is used. Therefore, it grants confidentiality, integrity, and authenticity of all retrieved health data from the Web service.

Two studies were performed: (1) a study evaluating which symmetric and asymmetric algorithm present the best performance in SapoFit in cooperation environment, and (2) a series of trials involving 35 people and 7 different mobile devices with SapoFit. This study evaluated the performance of the DE4MHA over the cooperation mechanisms.

### Study 1: Study of Cryptography Algorithms in an mHealth App Under a Cooperation Environment

#### Symmetric Algorithm

In order to choose the best suited symmetric encryption algorithm for the SapoFit app, performance experiments were conducted using 4 different encryption algorithms, namely AES, Triple Data Encryption Standard (3DES) [29], Rivest Cypher 4 (RC4) [30], and Blowfish [31], using the data size encryption as a performance metric.

#### Asymmetric Algorithm

Two options were considered in selecting an asymmetric algorithm to exchange session keys between mobile nodes. We tested RSA, which enables encrypting the session key before sending it, and Diffie-Hellman [32], which allows users to first share a secret, then generating a session key based on the shared secret. For our network scenario, these were the most suitable algorithms. Other encryption algorithms were considered in this study, such as the Elliptic Curve Cryptography (ECC) algorithm [33].

### Study 2: Performance evaluation of the DE4MHA

#### Performance Evaluation

**Figure 3:**
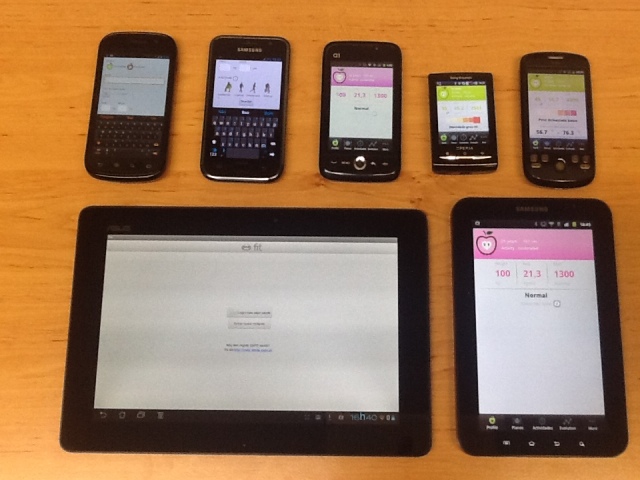
Mobile devices used for trials with the SapoFit mHealth app.

The performance evaluation study was carried out using 7 real mobile devices, which were used 7 times in 5 different days with a total of 35 different users who successfully tested the app. Figure 3 presents the 7 different mobile devices with different hardware and software used with the SapoFit mHealth app.

Cooperative nodes without network connection cooperated with each other through Bluetooth. The communication with the CWS was obtained through the Wi-Fi or Edge/3.5G/4G modules of the device. The CWS was developed with the help of Java Server Pages (JSP) technology, using the Representational State Transfer (REST) architecture. To serve the WS, the Apache Tomcat Web Server was used.

Non-cooperative cases were controlled and measured to a maximum of 4 to guarantee the minimum service performance in order to guarantee cooperation among nodes. Through cooperation, all the devices could use the mHealth app. However, uncooperative nodes directly affect the service delivery probability, service average delay, and the overall network performance. Performance metrics considered in this study were the service delivery probability (as percentages) and the average service delay (in seconds). We present a comparison of the performance of the mHealth app with and without the DE4MHA.

#### User Trials Evaluation of DE4MHA in Cooperative Environments

User trials were conducted within the University informatics department using 7 devices available for trials. Within 5 days, 5 different groups of 7 students selected randomly agreed to use and experiment the SapoFit app using the 7 devices available for trials. Users were constantly moving far away from each other. This mobility was necessary to test the network scenario, forcing network delays and disconnections. The cooperative strategy and the DE4MHA was ubiquitous to its user. Throughout the trials, users only experienced and used the obesity prevention services that SapoFit offered without any constraints or perception of any cooperation mechanism or encryption algorithm that was embedded in the mHealth app.

While conducting the experiments, almost every users asked if their information was being kept secure or not, clearly showing that they did not wanted their health information to be available or disclosed to unauthorized people, revealing privacy concerns. Another frequently raised question was the need to share Internet connectivity to other users.

We explained and justified that sharing and cooperating with other users was essential to obtain a better reputation to gain cooperation privileges over other nodes with worse reputation. Furthermore we demonstrated to them that SapoFit was not intrusive with other users’ personal data on the mobile device and only requested for SapoFit services.

**Figure 4:**
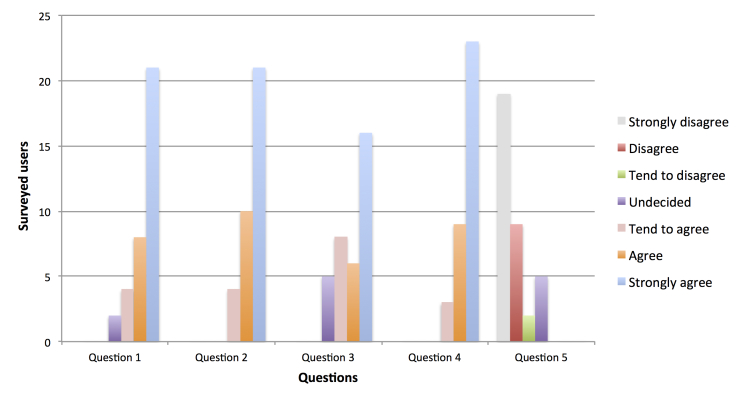
Results of the survey evaluating the main questions about the performance of the mHealth app with the proposed cooperation strategy and encryption solution.

After the experiments, the users completed a survey evaluating their experience. The questions are listed in Textbox 1 and the results in Figure 4.

**Survey questions.**

Without network connectivity, do the user always gets the required information?
Without network connectivity, does the required information get delivered in a comfortable time?
Understanding the implemented cooperation strategy and its benefits, are you willing to cooperate and share the device/network resources with other users?
With the encryption strategy applied to SapoFit, do you trust that your personal health information is secure?
Was the mobile device affected by app cooperation and encryption mechanisms (eg, broadband, battery, etc)?

## Results

### Symmetric Algorithm

**Figure 5:**
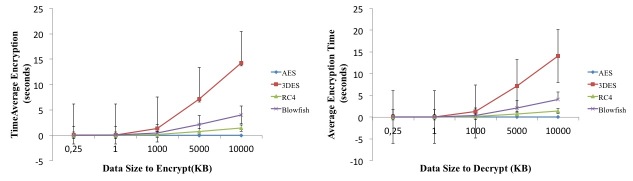
Comparison of encryption and decryption of symmetric algorithms (AES, 3DES, RC4, and Blowfish).

As seen in Figure 5, results showed that when the amount of data that needed to be encrypted increased, the encryption time (in seconds) also increased, as expected. When comparing small amounts of data, all 4 algorithms presented similar results. However, the AES algorithm presented better results, as there was a slower overall increase in encryption time in response to increased amount of data. The encryption time of the other three experimented algorithms grew exponentially when encryption data size exceeded 1000 kilobytes. The 3DES algorithm presented the worst encryption rate, encrypting 10,000KB of data in, on average, 14.3 seconds, presenting an average time of 4.58 seconds for multiple data size inputs (SD 6.17). With the same amount of data, the AES encryption time was only 0.0045 seconds, resulting in an average of 0.0035 seconds for the given data set (SD 0.00061 seconds). Decryption gave similar results (Figure 5). The average AES algorithm decryption time was 0.0038 seconds for 10,000KB of data and 0.0025 seconds on average for the working data-set (SD 0.001 seconds). Overall, the AES algorithm with a 128 bits key encryption was the most efficient algorithm for these network scenarios, when handling with both small and large amounts of data.

### Asymmetric Algorithm

Two options were considered for an asymmetric algorithm in order to exchange session keys between mobile nodes—the RSA and the Diffie-Hellman algorithms. The RSA encrypts the session key first before it gets sent, and Diffie-Hellman allows users to first share a secret, then generates a session key based on the shared secret.

Several experiments were performed with both algorithms with RSA presenting better encryption times than Diffie-Hellman, due to the high amount of computation needed by Diffie-Hellman and the low processing capacity of mobile devices.

### DE4MHA Performance Evaluation Results

In the presented scenario, all the devices had Bluetooth class 2 modules, but only 3 devices had Internet connectivity. Users without Internet connectivity had to use the integrated cooperation mechanisms in order to obtain the requested health information. When the number of uncooperative nodes increased, the service delivery probability decreased. The average service delay was also affected in the same mannor, as expected. Increased number of uncooperative nodes increased the average service delay.

**Figure 6:**
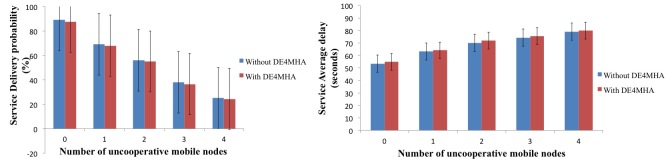
Service delivery probability and average service delay as a function of the number of uncooperative mobile nodes with and without the DE4MHA.

Figure 6 shows the results of the service delivery probability and the average service delay as a function of the number of uncooperative mobile nodes with and without the DE4MHA. The probability that a request was successfully answered as a function of uncooperative nodes in the network decreased with the increase of uncooperative mobile nodes. Taking into account both approaches, with and without DE4MHA, it was observed that DE4MHA presented a slightly worse result. The average service delay also grew when the number of uncooperative mobile nodes increased, as expected. The results of the DE4MHA were also slightly worse but practically insignificant. As can be observed, with 4 uncooperative nodes, the service average delay, with and without DE4MHA, was about 30.78 and 29.77 seconds, respectively.

## Discussion

### Findings

Our main goal was to propose and construct a security encryption/decryption based solution in a cooperative environment for mHealth apps. We aimed to ensure data confidentiality, integrity, and authenticity. Privacy is a top priority issue in mHealth services and apps that deal with user sensitive information. On mHealth apps, several security issues must be considered, such as personal information management, secondary use of personal information, improper use of personal information, and errors with stored personal information. Therefore, cryptographic mechanisms can be seen as a solution to guarantied data confidentiality and protection [34].

In a mobility and cooperative environment with constant health data being forwarded and retrieved, studying and developing security mechanisms become crucial. Several experiments were conducted, involving 35 different users, to check if they could distinguish the app running with and without the DE4MHA embedded. Through the trials, we concluded that users could not tell which app had the DE4MHA embedded mainly because the time response taken to obtain the user health information was nearly the same as without DE4MHA. The DE4MHA was implemented in a ubiquitous manner so users were able to keep using the app without noticing changes or presence of any cryptography mechanisms.

### Limitations

There were several limitations to the study. The main limitation was applying security on mobile devices due to the low processor capacity compared with personal computers (PCs), though tremendous improvements in this area have been made, with a few mobile devices capable of competing with traditional PCs. This improvement allowed us to test several security algorithms to address the issues of confidentiality (AES, RC4, 3DES, and Blowfish), integrity (MD5 and SHA1), and authenticity (RSA with MD5 and DSA with SHA1) in order to verify which combination had a better performance in a mobile environment.

During the experiments, some users without Internet connectivity who wanted to obtain health information were in constantly moving further away from other users. Although the cooperation mechanism was embedded, users that were beyond the range of 10 meters (the maximum range for Bluetooth class 2 modules) could not obtain the desired health information. Another limitation, though not related to security, was with regard to the number of uncooperative nodes (mobile nodes that may not want to cooperate or they may not have cooperation mechanisms embedded), compromising service response probability.

### Conclusion

This paper proposed a data encryption solution for mobile health apps, called DE4MHA. The data encryption algorithm DE4MHA with cooperation mechanisms in mobile health allow users to safely obtain health information with the data being carried securely. These security mechanisms did not deteriorate the overall network performance and the app, maintaining similar performance levels as without the encryption. More importantly, it offers a robust and reliable increase of privacy, confidentiality, integrity, and authenticity of their health information. Although it was experimented on a specific mHealth app, SapoFit, both DE4MHA and the cooperation strategy can be deployed in other mHealth apps.

## Abbreviations

3DES: Triple Data Encryption Standard
ACT: achieved cooperation time
AES: Advanced Encryption Standard
BMI: body mass index
BMR: basal metabolic rate
CWS: cooperative Web service
DE4MHA: data encryption solution for mobile health apps
EHR: electronic health record
HTTPS: Hypertext Transfer Protocol Secure
ICT: information and communication technologies
JSP: Java Server Pages
RC4: Rivest Cipher 4
REST: Representational State Transfer
RSA: Rivest, Shamir, Adleman
SOAs: service oriented architectures
SSL: Secure Socket Layer
WS: Web service
